# The effectiveness of simulation-based education combined with peer-assisted learning on clinical performance of first-year medical residents: a case-control study

**DOI:** 10.1186/s12909-023-04798-w

**Published:** 2023-11-12

**Authors:** Taku Murakami, Akira Yamamoto, Hideharu Hagiya, Mikako Obika, Yasuhiro Mandai, Tomoko Miyoshi, Hitomi Kataoka, Fumio Otsuka

**Affiliations:** 1 Department of General Medicine, Dentistry and Pharmaceutical Sciences, Okayama University Graduate School of Medicine, 2-5-1 Shikata-cho, Kita-ku, Okayama, Japan; 2 Department of Hematology and Oncology, Okayama University Hospital, 2-5-1 Shikata-cho, Kita-ku, Okayama City, Okayama Japan; 3 Department of Emergency Medicine, The JIKEI University, 3-25-8 Nishi-Shinbashi, Minato-ku, Tokyo, Japan; 4 Diversity Enhancement Center, Okayama University Hospital, 2-5-1 Shikata-cho, Kita-ku, Okayama City, Okayama Japan

**Keywords:** Medical Education, Educational Measurement, Simulation Training, Peer Group, Emergency Medicine, Internship and residency, Curriculum, Personal satisfaction, Case-control studies

## Abstract

**Background:**

Simulation-based education and peer-assisted learning (PAL) are both known as useful educational methods. Previous research has reported that combining these two methods are effective for training medical residents in short-term evaluation. This study was aimed to evaluate the middle- to long-term effects of simulation-based education combined with PAL on the performance of medical residents during emergency department duties.

**Methods:**

This study was designed as a case-control study and conducted over three years at Okayama University Hospital in Japan. Postgraduate-year-one medical residents were assigned to three groups: a simulation group that received simulation-based education, a lecture group that received traditional lecture-based education, and a control group that received no such prior trainings. Prior training in emergency department duties using PAL was performed as an educational intervention for the simulation and lecture groups during the clinical orientation period. The residents’ medical knowledge was assessed by written examinations before and after the orientation. The performance of residents during their emergency department duties was assessed by self-evaluation questionnaires and objective-assessment checklists, following up with the residents for three months after the orientation period and collecting data on their 1st, 2nd, and 3rd emergency department duties. All the datasets collected were statistically analyzed and compared by their mean values among the three groups.

**Results:**

A total of 75 residents were included in the comparative study: 27 in the simulation group, 24 in the lecture group, and 24 in the control group. The simulation and lecture groups obtained significantly higher written examination scores than the control group. From the self-evaluation questionnaires, the simulation group reported significantly higher satisfaction in their prior training than the lecture group. No significant differences were found in the emergency department performance of the residents among the three groups. However, when evaluating the improvement rate of performance over time, all three groups showed improvement in the subjective evaluation, and only the simulation and lecture groups showed improvement in the objective evaluation.

**Conclusion:**

Simulation-based education combined with PAL is effective in improving the knowledge and satisfaction of medical residents, suggesting the possibility of improving work performance during their emergency department duties.

## Background

Simulation-based education is a highly effective method for the acquisition of new skills [1–3]. Specifically, in the field of emergency medicine, numerous studies have confirmed the effectiveness of simulation-based education in providing learners with a safe training environment [4, 5]. Peer-assisted learning (PAL) is another effective approach for training young doctors in clinical practices [6–8]. Our previous research has shown that the combination of simulation-based education and PAL yields superior learning outcomes compared to traditional lecture-based education, as evidenced by short-term evaluations [7]. However, the medium- and long-term effects of simulation-based education in clinical settings have not been revealed [7]. As of October 2023, we searched PubMed database for relevant keywords. Although there are 7,671 results for studies with the keywords “simulation” and “emergency medicine” and 1,413 results for studies with the keywords “peer-assisted learning”, only 18 studies contain all three keywords and no case-control studies have been reported on simulation-based education combined with PAL as prior training for emergency department duties. Revealing the educational outcomes of combining simulation-based education and PAL for training medical residents can provide valuable insights into the curriculum development for future medical training programs. This study was designed as a case-control study to assess the effectiveness of integrating simulation-based education and PAL as prior training for postgraduate-year-one (PGY-I) medical residents in performing their emergency department duties.

## Methods

This case-control study was conducted over three years from 2016 to 2018, at Okayama University Hospital in Japan, focusing on PGY-I medical residents. The PGY-I residents were divided into three groups: a simulation group receiving simulation-based education as prior training, a lecture group receiving traditional lecture-based education as prior training, and a control group with no prior training. The prior training was conducted during the orientation period for PGY-I prior to their clinical practice. The control group, which did not receive any prior training but attended the orientation program, was established to compare the impact of prior training among the groups. In addition, the simulation and lecture groups were compared to evaluate the educational methods of the prior training performed in each group. All PGY-I residents were followed up until the end of their two-year medical residency program to ascertain the department they chose for their future specialties. The eligibility criteria for participation required the submission of a consent form. Participants with a response rate of less than 50% on the required questionnaires and checklists were excluded due to insufficient data. The residents were divided into the three groups based on the order of their names in the Japanese syllabary.

**Fig. 1:**
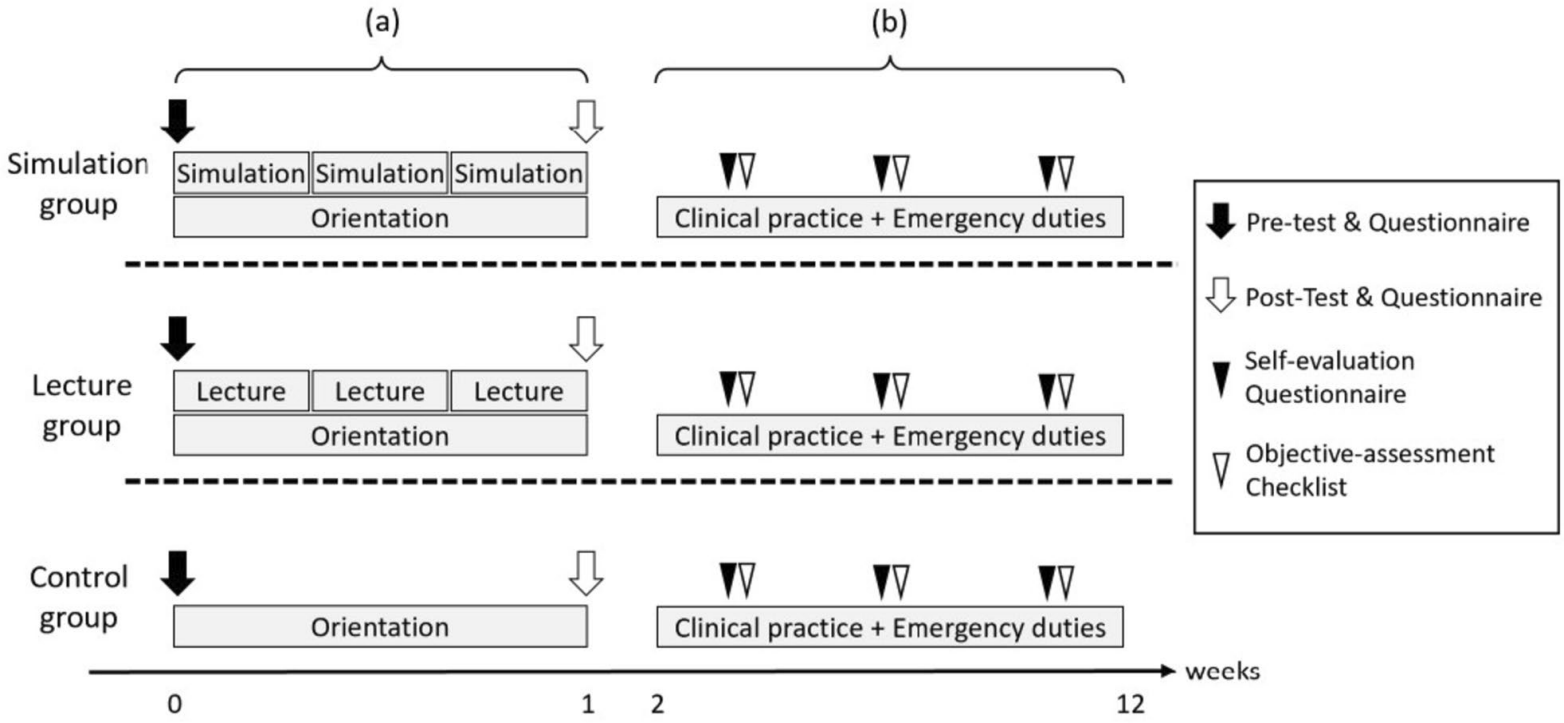
Research design and structure. **a **Performed tests and questionnaires before and after the 2-week clinical orientation. For the simulation and lecture group, three sessions of simulation training and lecture training were held during the orientation period. **b**  During the clinical practice, self-evaluation questionnaires and objective-assessment checklists were used to assess residents after each performance of emergency department duties

Our previous study performed by Yamamoto et al. [7] found that the combination of simulation-based education and PAL has a significantly positive effect on the learning outcomes of medical residents in a short-term evaluation. However, medium- and long-term evaluations of the learning effects of simulation-based education combined with PAL and its effects on actual clinical practices have not been fully investigated. In this study, we analyzed the effects of prior trainings using simulation-based education and PAL on the emergency department duties of PGY-I residents. We utilized the PAL method demonstrated in the previous study [7] where postgraduate-year-two (PGY-II) residents taught PGY-I residents and developed case studies for prior training sessions. The case study scenarios were designed as emergency medicine cases and were supervised by specialized staff (Murakami, Yamamoto, and Mandai) trained to provide simulation-based education at the University of Hawaii (John A. Bunrs School of Medicine, Sim Tiki Simulation Center University). Three case study scenarios –acute myocardial infarction, multiple traumas, and aspiration pneumonia– were prepared as typical cases encountered by medical residents in their emergency department duties, and all case studies were evaluated for the level of difficulty, validity, accuracy, and effectiveness through actual simulation and lecture sessions beforehand [7, 9, 10]. The duration of the prior training for the simulation and lecture groups was adjusted to be the same length in order to avoid any bias due to differences in study time. Prior clinical training for the simulation and lecture groups was performed in addition to the clinical orientation for all PGY-I residents to participate; for the control group, only clinical orientation was performed, and no prior training was performed. To avoid potential bias, the assigned groups of each PGY-I resident were not revealed to other participants, and the residents were strictly prohibited from sharing any information acquired during prior training. All PGY-I residents were given a questionnaire and a pre-test in the form of a written examination at the beginning of the clinical orientation period to ascertain their demographics and academic performance. The simulation, lecture, and control groups received simulation-based education as prior training, lecture-based education as prior training, and no prior training, respectively. After the clinical orientation and educational interventions were completed, all PGY-I residents were given another questionnaire and a post-test in the form of a written examination to re-evaluate their academic performance (Fig. 1a). Questionnaire items of the written examinations were in common in pre-test and post-test, being composed of 24 multiple choice questions, four short-answer questions, and one essay question. This was made up to evaluate the knowledge of emergency cases of acute myocardial infarction, multiple traumas, and aspiration pneumonia.

The performance of PGY-I residents during their emergency department duties was evaluated by following up with the residents for three months after the clinical orientation period and collecting data on their 1st, 2nd, and 3rd emergency department duties. To assess resident performance during emergency department duties, self-evaluation questionnaires were administrated to the PGY-I residents to collect subjective data, and objective-assessment checklists were provided to the PGY-II residents who worked with the relevant PGY-I residents to collect objective data. The validity of questionnaires and checklists for assessing trainee performance was reported by Boynton et al. [11] and Artino et al. [12]. For our study, each item of the self-evaluation questionnaire and objective-assessment checklist was rated on a 10-point scale. The self-evaluation questionnaire included eight elements: medical history, assessment of the patient’s general condition, physical examination, laboratory testing, treatment, differential diagnosis, follow-up assessment, and work stress level. Additionally, residents in the simulation and lecture groups were asked to rate the extent to which they found their prior training during the orientation period useful for their emergency department duties on a 10-point scale. The elements of the objective-assessment checklist were designed to be the same as the eight elements of the self-evaluation questionnaire so that the results could be easily compared. To avoid bias, PGY-II residents who evaluated PGY-I residents were not informed about which group they were assigned to (Fig. 1b). This study firstly evaluated the short-term effects of simulation-based education combined with PAL as prior training for emergency medicine by written examinations, which was performed shortly after the training. Secondly, we evaluated the middle-term effects by assessing the residents’ clinical performances during their first emergency department duties, which was performed approximately one to two weeks after the prior training. Finally, we evaluated the long-term effects by following up the residents for three months after the prior training and continuing their assessment during emergency department duties.

The results of the pre-test and post-test during the orientation period, as well as the results of each element of the self-evaluation questionnaire and objective-assessment checklist for emergency department duties, were statistically analyzed and compared by their means among the three groups. IBM SPSS Statistics 26™ and Prism 9 for Mac™ (Version 9.5.0) was used for statistical analyses. Chi-square tests were applied to categorical variables such as resident demographics. Paired t-tests were applied to compare continuous variables between the pre-tests and post-tests within the individual groups, and one-way analysis of variance (ANOVA) were applied to compare continuous variables of the pre-tests and post-tests among all three groups. For the evaluation of emergency department duties, student t-tests were applied to compare continuous variables between the simulation group and the lecture group. One-way ANOVA were applied to compare continuous variables obtained from each emergency department duties among all three groups, and repeated measures ANOVA were applied to compare continuous variables obtained from the multiple emergency department duties within the individual groups. No information of members and contents of the prior trainings were given to evaluators or fellow residents to avoid any potential confounders and bias as possible. For the simulation and lecture groups, in which prior training was provided as an educational intervention, principal component analysis (PCA) was performed for all elements of the self-evaluation questionnaire and the objective-assessment checklist. The study was conducted in accordance with the appropriate procedures of the research program at the Okayama University Hospital, and permission was obtained from the ethics committee (No. 1602-039). To eliminate any inequities in the initial clinical training of medical residents, equal opportunities were ensured for PGY-I residents to attend both simulation- and lecture-based prior training after the evaluation period of the study upon request.

## Results

**Fig. 2:**
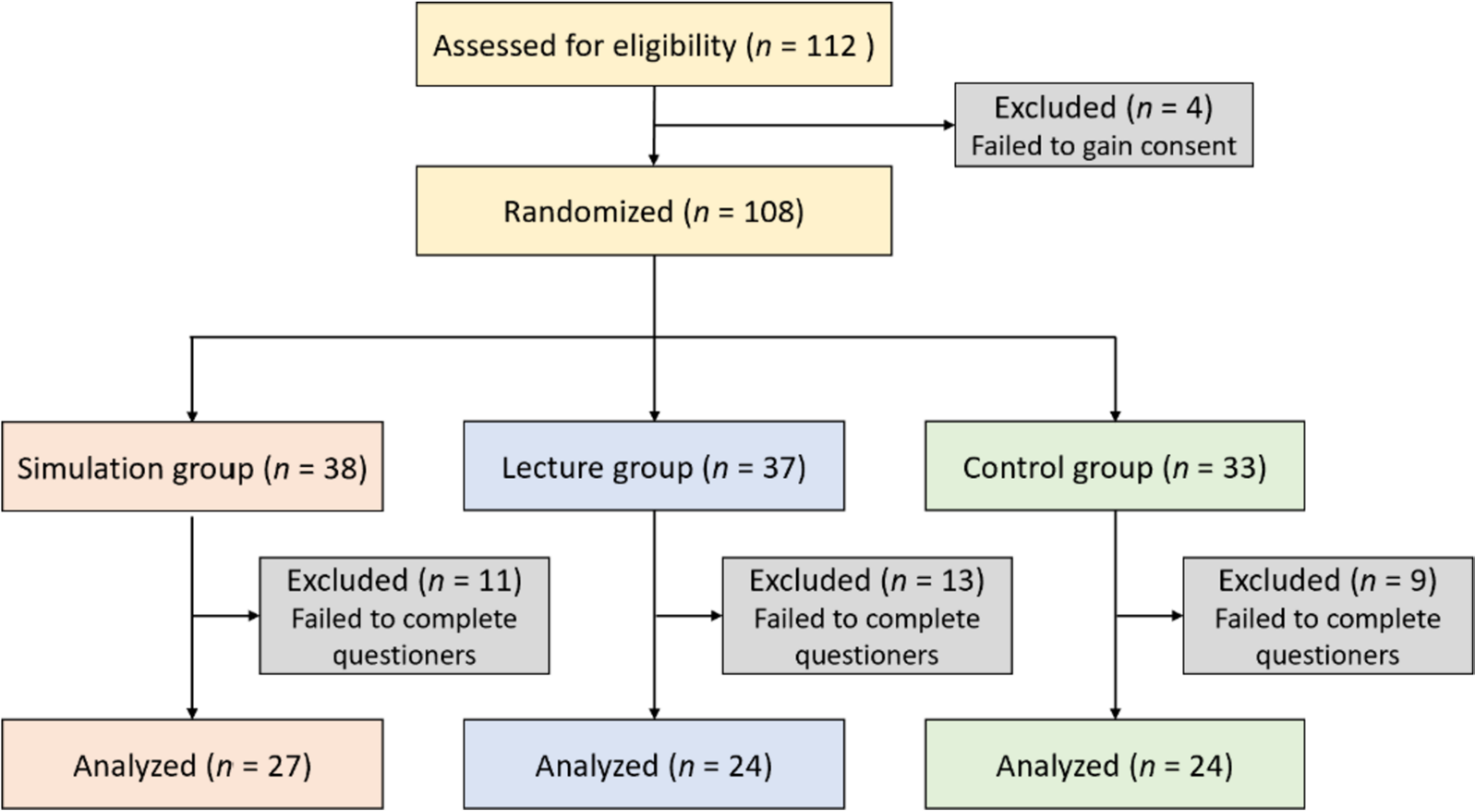
Inclusion and exclusion criteria.  A total of 108 first-year medical residents were randomized and divided into three groups, and 75 were analyzed: the simulation group (*n* = 27), lecture group (*n* = 24), and control group (*n* = 24)

**Fig. 3:**
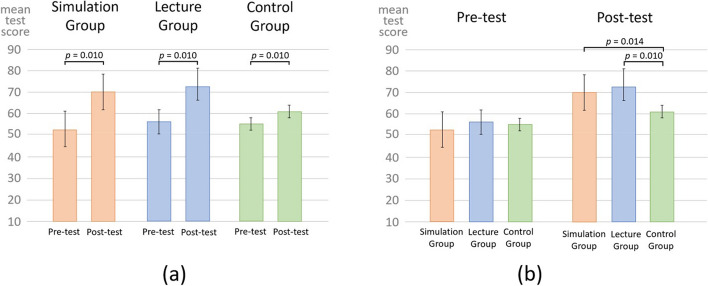
Evaluation of the pre-test and post-test scores. **a **Post-test scores were significantly higher than pre-test scores in all three groups. **b** Post-test scores were significantly higher in the simulation and lecture groups than in the control group.  Statistically analyzed by paired t-test and one-way ANOVA. Whiskers indicate standard deviation values

A total of 75 residents were included in the comparative study: 27 in the simulation group, 24 in the lecture group, and 24 in the control group (Fig. 2). No significant differences were found between the three groups in the demographics of the residents, as obtained using a questionnaire during the clinical orientation period (Table 1a). In addition, no significant differences were found between the groups in the pre-test results during the orientation period (Table 2; Fig. 3a, b). Furthermore, no significant differences were found between the groups in the future department of choice obtained from a follow-up survey performed after the initial clinical training of the PGY-I residents (Table 1b).

**Table 1:** Demographics of the participants

Demographics from the pre orientation questionnaire	Number of residents (%)		
	Simulation	Lecture	Control
Male	15 (56)	15 (62)	18 (75)
Female	12 (44)	9 (38)	6 (15)
Experienced simulation-based education in medical school	22 (82)	16 (67)	22 (92)
Experienced being a tutor of simulation-based education	3 (11)	3 (13)	2 (8)
Willing to instruct simulation-based education	14 (58)	11 (46)	14 (58)
Belonged to a study group or training club in medical school	11 (41)	7 (29)	8 (33)
Graduated from Okayama University medical school	10 (37)	11 (46)	10 (42)
Graduated from other university medical schools	17 (63)	13 (54)	14 (58)
(a) Demographics of PGY**-**I residents based on the pre orientation questionnaire collected at the beginning of the clinical orientation Statistically analyzed by Chi-square test			

Demographics from the post medical training questionnaire	Number of residents (%)		
	Simulation	Lecture	Control
Internal Medicine Department for future specialty	11 (41)	10 (42)	12 (50)
Surgical Department for future specialty	14 (52)	13 (54)	10 (42)
(b) Demographics of PGY-I residents based on the post medical training questionnaire collected after the completion of the initial medical training. Statistically analyzed by Chi-square test

**Table 2:** Knowledge evaluation using pre-test and post-test scores

	Pre-test		Post-test		
	Mean	SD	Mean	SD	Difference
Simulation group	52.7	11.8	69.9	9.3	17.2
Lecture group	55.9	14.0	72.6	10.9	16.8
Control group	55.1	13.1	61.4	10.6	6.3

Mean, standard deviation (SD), and score differences in the pre**-** and post**-**tests for each group

A comparison between the pre-test and post-test scores of the three groups during the orientation period showed a significant increase in the mean score (simulation group, pre-test score 52.7 [11.8] vs. post-test score 69.9 [9.3], *p* = 0.010; lecture group, pre-test score 55.9 [14.0] vs. post-test score 72.6 [10.9], *p* = 0.010; control group, pre-test score 55.1 [13.1] vs. post-test score 61.4 [10.6], *p* = 0.010) (Fig. 3a). In the pre-test, there were no significant differences in the mean scores among the three groups. For the post-test, the simulation and lecture groups showed a significant increase in the mean scores compared to the control group (simulation group post-test score 69.9 [9.3] vs. control group post-test score 61.4 [10.6], *p* = 0.014; lecture group post-test score 72.6 [10.9] vs. control group post-test score 61.4 [10.6], *p* = 0.010). A comparison between the simulation and lecture groups showed no significant differences in the mean scores of the post-test (Fig. 3b).

**Fig. 4:**
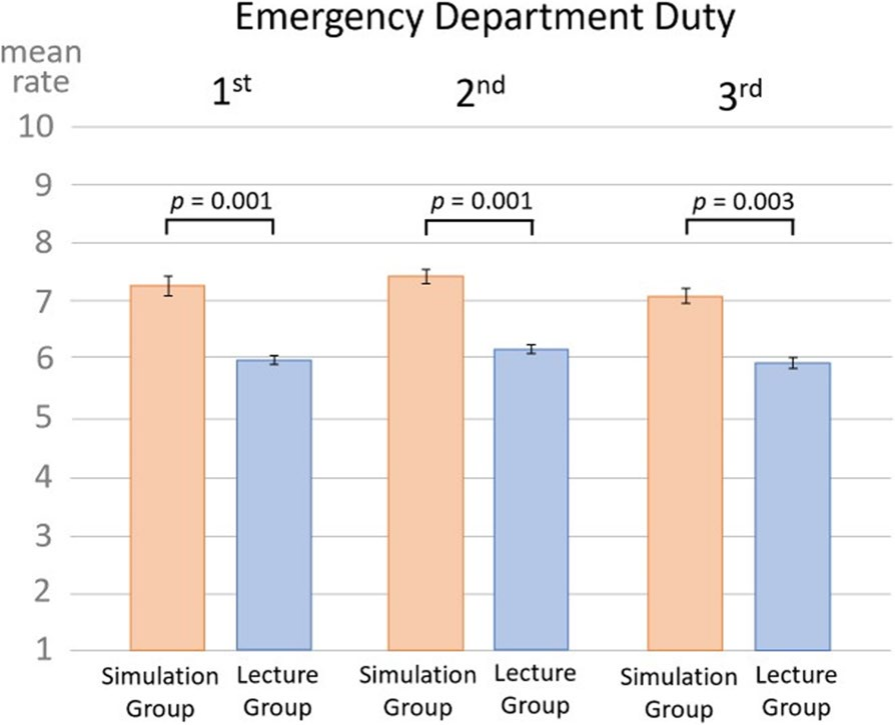
Usefulness of the prior training.  Simulation group finds their prior training more useful compared to the Lecture group.  Statistically analyzed by Student’s t-test  (1st emergency department duty: mean rate of the simulation group 7.26, and the lecture group 4.96, *p*  = 0.001. 2nd emergency department duty: mean rate of the simulation group 7.41, and the lecture group  5.16, *p * = 0.001. 3rd emergency department duty: mean rate of the simulation group 7.10, and the lecture group 4.94, *p*  = 0.003)  Whiskers indicate standard deviation values

**Fig. 5:**
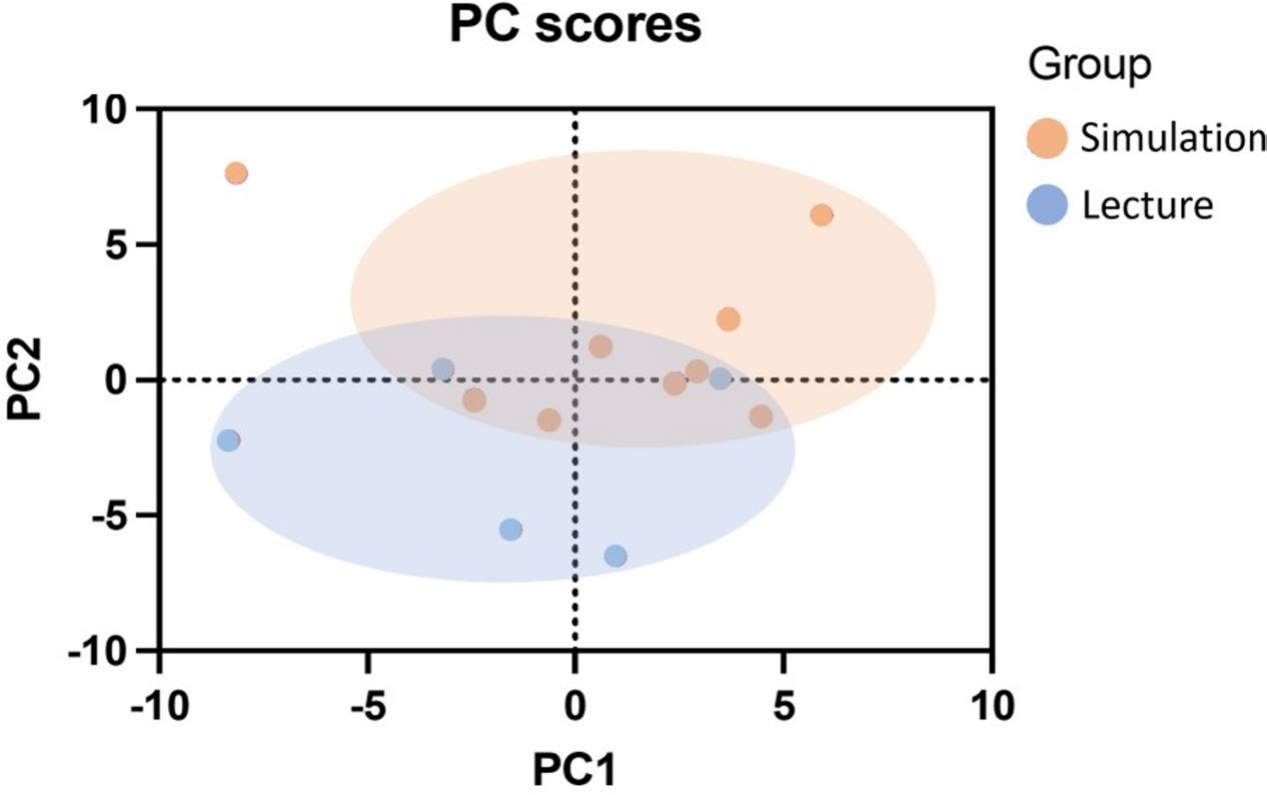
Principal component analysis. The principal component (PC) analysis of the self-evaluation questionnaire and the objective-assessment checklist of all emergency department duties for both the simulation and lecture groups shows the tendency of the simulation group to have higher rate on the self-evaluation questionnaire than the lecture group. The first 2 components (contributing 47.89% of variance) were plotted

For evaluating the efficacy of prior training for emergency department duties, the results of the self-evaluation questionnaire were compared between the simulation and lecture groups. The simulation group showed a significantly higher rating for assessing whether prior training was useful for emergency department duties than the lecture group for all 1st, 2nd, and 3rd emergency department duties (1st emergency department duty, simulation group rate 7.3 [1.8] vs. lecture group rate 5.0 [2.3], *p* = 0.001; 2nd emergency department duty, simulation group rate 7.4 [2.1] vs. lecture group rate 5.2 [1.9], *p* = 0.001; and 3rd emergency department duty, simulation group rate 7.1 [1.9] vs. lecture group rate 4.9 [1.9], *p* = 0.003) (Fig. 4). Moreover, for evaluating the impact of prior training, PCA of the elements of the self-evaluation questionnaire and the objective-assessment checklist revealed a tendency for the simulation group to have a higher rating on the self-evaluation questionnaire than the lecture group (Fig. 5).

**Fig. 6:**
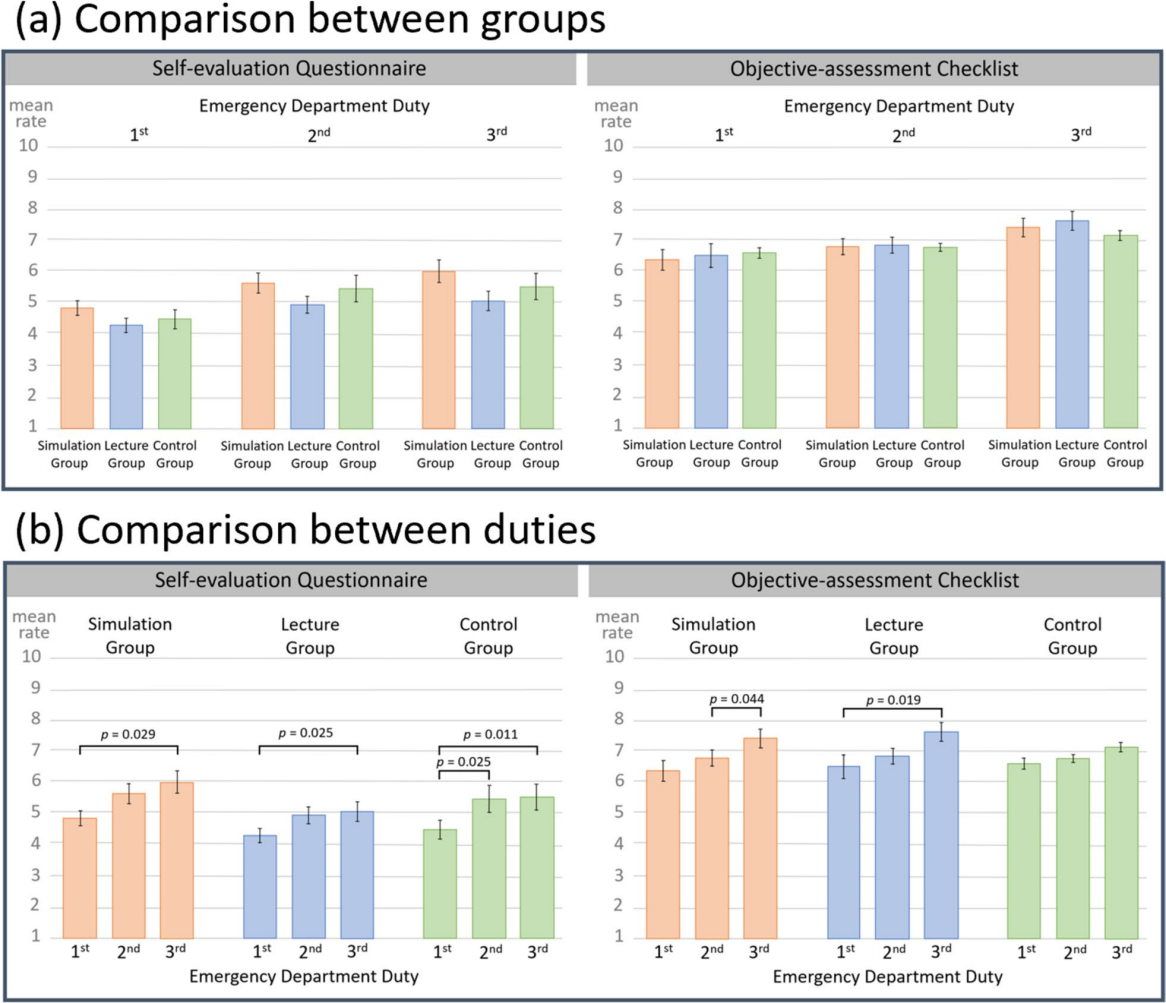
Comparison of the results of the self-evaluation questionnaire and objective-assessment checklist. **a** One-way ANOVA showed no significant differences among the three groups in the results of the questionnaire and checklist during the 1st to 3rd emergency department duties. **b** Repeated measures ANOVA showed that all three groups have significantly higher self-evaluation questionnaire ratings throughout the 1st to 3rd emergency department duties. The simulation and lecture groups have significantly higher objective-assessment checklist ratings whereas the control group showed no significant difference throughout the 1st to 3rd emergency department duties.  Whiskers indicate standard deviation values

No significant differences were observed among the three groups in the mean rate of the self-evaluation questionnaire and that of the objective-assessment checklist for evaluating the clinical performance of PGY-I residents during emergency department duties (Fig. 6a). However, evaluating the improvement rate of the resident performances over time by comparing the results of the 1st, 2nd, and 3rd emergency department duties through a repeated measures ANOVA, all three groups showed a significant increase in the mean rate of the self-assessment questionnaire. The simulation group showed significant improvement between the 1st and 3rd emergency department duties (1st emergency department duty rate 5.0 [1.7] vs. 3rd emergency department duty rate 6.0 [1.7], *p* = 0.029); the lecture group showed significant improvement between the 1st and 3rd emergency department duties (1st emergency department duty rate mean 4.3 [1.3] vs. 3rd emergency department duty rate 5.1 [1.4], *p* = 0.025); and the control group showed significant improvement between 1st and 2nd (1st emergency department duty rate 4.5 [1.1] vs. 2nd emergency department duty rate 5.5 [1.3], *p* = 0.011) as well as 1st and 3rd emergency department duties (1st emergency department duty rate 4.5 [1.1] vs. 3rd emergency department duty rate 5.5 [1.1], *p* = 0.004). From the results of the objective-assessment checklist, the simulation group showed significant improvement between the 2nd and 3rd emergency department duties (2nd emergency department duty rate 6.8 [1.1] vs. 3rd emergency department duty rate 7.4 [0.9], *p* = 0.044) through a repeated measures ANOVA and post-hoc comparison (Tukey), and the lecture group showed significant improvement between the 1st and 3rd emergency department duties (1st emergency department duty rate 6.6 [1.4] vs. 3rd emergency department duty rate 7.6 [1.0], *p* = 0.019), whereas the control group showed no significant improvement in performance during emergency department duties over time (Fig. 6b).

Pearson’s correlation test was used to evaluate the association between the demographics of the PGY-I residents, the scores of the written examinations, and the mean rates of performance during the emergency department duties, but the results showed no significant correlation. Additionally, one-way ANOVA was used to compare the improvement rate of emergency department performance among the three groups. Stratified analyses were performed by independently evaluating residents with average or higher scores on the written examinations and those with lower scores; however, no significant trend was obtained (data not shown).

## Discussion

All three groups showed significant improvement in the mean scores on the post-test compared with the pre-test. However, comparing the improvement rate of the mean test scores, simulation and lecture groups showed significantly greater improvement than the control group. These results are consistent with previous reports indicating that educational interventions during the clinical orientation period can improve PGY-I residents’ knowledge [13–15]. In addition, a previous study showed that PAL combined with simulation-based education has a better learning effect than lecture-based education in a short-term evaluation [7]. This study showed no significant differences in test scores between the simulation and lecture groups. However, the groups that received prior clinical training showed a significant increase in mean test scores compared to the group that did not, which is consistent with previous reports that educational intervention based on PAL has positive effects on improving test scores [13, 15].

Previous studies have shown that PAL has greater learning effects than conventional education; in particular, PAL combined with simulation-based education has been suggested to be highly effective [6–8]. In this study, PAL was employed during the orientation period, and both the simulation and lecture groups performed significantly better on the written examination than the control group, which is consistent with a previous study by Yamamoto et al. [7], indicating the short-term efficacy of PAL. Furthermore, the evaluation of the emergency department performance of PGY-I residents showed a significant increase in the improvement rate over time for the objective assessment of the simulation and lecture groups whereas the control group showed no significant changes, suggesting that prior training based on PAL can improve performance during emergency department duties.

A previous study adopted PAL with PGY-II residents teaching PGY-I residents and noted that, as one of the advantages of PAL, young learners felt more comfortable asking questions compared to cases in which more experienced doctors were teaching [7]. The PAL adopted in our study may have created an environment in which the PGY-I residents were encouraged to ask questions, thus promoting effective learning. However, this study did not investigate the effects of experienced doctors teaching PGY-I residents, nor did it investigate the effects of PAL on PGY-II residents; further studies are needed. Examining the correlation between PGY-I residents’ demographics, test scores, and performance during their emergency department duties showed no consistent trends. Therefore, we believe that prior training through simulation-based learning based on PAL has a universal educational effect regardless of resident characteristics.

Comparing the effects of prior training on the simulation and lecture groups, there were no significant differences in the test scores during the orientation period or in the performance during emergency department duties. However, when asked in the self-evaluation questionnaire whether the PGY-I residents felt that their prior training was useful in the performance of their emergency department duties, the simulation group reported significantly higher satisfaction than the lecture group and reported that they felt their prior training was effective. Furthermore, PCA of the self-evaluation questionnaire and the objective-assessment checklist during emergency department duties showed a tendency for the simulation group to have higher rates on the self-evaluation questionnaire than the lecture group. These results suggest that prior training in simulation-based education based on PAL may be more satisfactory than lecture-based education and may lead to higher self-evaluation in clinical practice. Ten et al. [16] also reported satisfaction with simulation-based learning, and the results of our study are consistent with previous findings in the context of simulation-based education having greater satisfaction than lecture-based education for prior training in emergency department duties.

There were no significant differences among the three groups in PGY-I resident performance during emergency department duties according to the results of the self-evaluation questionnaires and the objective-assessment checklists. However, by evaluating the improvement rates of PGY-I resident performance during their emergency department duties, the simulation and lecture groups showed significantly higher scores compared to the control group according to the results of the objective-assessment checklists. Thus, these results suggest that prior training based on PAL may have a long-term beneficial effect on the performance improvement rate of PGY-I residents during emergency department duties. Although the evaluation period in this study was only three months, the observed increase in the improvement rate of performance during emergency department duties for the groups that received prior training suggests the possibility of further improvement in clinical performance over time, and further long-term evaluations are needed in the future. While previous studies have reported simulation-based education to be more effective than lecture-based education [17], this study found no significant differences between the learning effects of simulation-based education and lecture-based education for written examinations and emergency department duty performance. However, simulation-based education was found to be more effective for improving self-evaluation and increasing satisfaction for residents. Therefore, conducting prior training in emergency department duties via simulation-based education combined with PAL may not only lead to improving the work performance of the residents but may also contribute to improving resident motivations toward better clinical practices. A previous study also reported an increase in self-learning time as a short-term effect of simulation-based education combined with PAL [7]. As a long-term effect, such changes in behavior and awareness are expected to improve work performance in PGY-I residents.

The most notable limitation of this study is the continuity and the repeatability of simulation-based education as prior training for emergency medicine. Also, the fact that the study design did not completely restrict residents’ interactions and individual learnings may be a potential source of bias. In addition, this study was conducted as a single-center study limiting the number and variety of the residents evaluated, which may be a possible restriction for generalization of the results. A possible reason for the lack of significant differences in performance during emergency department duties among the three groups is that no additional educational intervention was provided after the initial orientation sessions. Previous studies have shown that the duration of the learning effects of simulation-based education is limited over time, and repeated educational interventions are necessary for the retention of learning contents [18, 19]. To achieve significant improvements in work performance, multiple interventions may be necessary after starting the emergency department duties to avoid long intervals between the training and actual clinical practices. For future research, study design with repeated educational intervention is recommended to further investigate the long-term impact of simulation-based education. Another possible factor that may have influenced the results of this study is that, although the assignment of PGY-I and PGY-II residents into groups was anonymous to avoid any bias, because the control group received no prior training, incentives to study privately may have been promoted for the residents assigned to the control group because of competitive consciousness among the residents. In addition, this study was conducted at a university hospital in Japan, where advanced and specialized medical services are provided, and medical practices, as well as the types of diseases and patients, differ from those of primary care hospitals. Although the case study scenarios for prior training in our study were developed as common cases that medical residents encounter during emergency department duties, adjusting the case study scenarios considering the role and characteristics of the institutions where prior training is provided may result in a more effective educational investigation.

Ensuring financial costs as well as faculty time and education is important for preparing prior trainings, and further evaluations are needed [20, 21]. In this study, no additional financial costs were necessary because preexisting facilities, including the simulator, were utilized for prior training. The time and human costs necessary for providing prior training were reduced by accumulating case study scenarios and educational programs. Thus, for institutions where simulators and facilities for simulation-based education are already available, simulation-based education may be a decent choice for the means of prior training in emergency medicine, especially in terms of medical residents’ satisfaction.

## Conclusions

The objective of this study was to assess the middle- to long-term effects of prior training using different educational methods combined with PAL through a case-control trial design. The results indicated that both simulation-based and lecture-based education combined with PAL enhanced the clinical knowledge of medical residents. Prior training in the form of simulation-based or lecture-based education demonstrated the potential to enhance the clinical performance of medical residents during emergency department duties. Additionally, when comparing simulation-based and lecture-based education, simulation-based education was associated with a significantly higher level of satisfaction among medical residents. This study was conducted on a small scale at a university hospital in Japan. Further large-scale and long-term studies in various medical institutions are needed to provide a more comprehensive evaluation.

## Data Availability

The datasets used and/or analyzed during the current study are available from the corresponding author on reasonable request.

## Abbreviations

PAL: Peer-assisted learning
PGY-I: Postgraduate-year-one
PGY-II: Postgraduate-year-two
ANOVA: Analysis of variance
PCA: Principal component analysis
SD: Standard deviation
